# Errors in determination of net survival: cause-specific and relative survival settings

**DOI:** 10.1038/s41416-020-0739-4

**Published:** 2020-02-10

**Authors:** Chloe J. Bright, Adam R. Brentnall, Kate Wooldrage, Jonathon Myles, Peter Sasieni, Stephen W. Duffy

**Affiliations:** 1 National Cancer Registration and Analysis Service, Public Health England, London, UK; 2 Wolfson Institute of Preventive Medicine, Barts and The London School of Medicine and Dentistry, Queen Mary University of London, London, UK; 3 Department of Surgery and Cancer, Imperial College London, London, UK; 4 School of Cancer and Pharmaceutical Sciences, Faculty of Life Sciences & Medicine, King’s College London, London, UK

**Keywords:** Cancer epidemiology, Epidemiology

## Abstract

**Background:**

Cause-specific and relative survival estimates differ. We aimed to examine these differences in common cancers where by possible identifying the most plausible sources of error in each estimate.

**Methods:**

Ten-year cause-specific and relative survival were estimated for lung, breast, prostate, ovary, oesophagus and colorectal cancers. The cause-specific survival was corrected for misclassification of cause of death. The Pohar-Perme relative survival estimator was modified by (1) correcting for differences in deaths from ischaemic heart disease (IHD) between cancers and general population; or (2) correcting the population hazard for smoking (lung cancer only).

**Results:**

For all cancers except breast and prostate, relative survival was lower than cause-specific. Correction for published error rates in cause of death gave implausible results. Correction for rates of IHD death gave slightly different relative survival estimates for lung, oesophagus and colorectal cancers. For lung cancer, when the population hazard was inflated for smoking, survival estimates were increased.

**Conclusion:**

Results agreed with the consensus that relative survival is usually preferable. However, for some cancers, relative survival might be inaccurate (e.g. lung and prostate). Likely solutions include enhancing life tables to include other demographic variables than age and sex, and to stratify relative survival calculation by cause of death.

## Background

Cancer survival is most often measured using estimated net survival, which under certain assumptions may be interpreted as the probability of surviving cancer in the absence of any other causes of death.^1^ For estimation of net cancer survival, researchers generally use either cause-specific survival or relative survival analysis methods. Cause-specific survival analysis takes a specific cause of death as the end point of interest and all the other causes of death are treated as independent censoring. Population-based studies that use cause-specific survival normally ascertain cause of death from the death certificate. Relative survival does not require information on cause of death in the cohort of cancer patients; instead it is calculated as the overall observed survival in the cohort relative to that expected in a general population without the cancer of interest. Relative survival often uses lifetables (normally by sex and age) from the general population to calculate the expected survival. Relative survival was calculated as a ratio of observed to expected survival in the past.^2^ It is more common now to calculate it based on subtraction of the expected population hazard rate for death from all causes from the observed hazard of all-cause death in the cancer cohort.^3^ Such methods are still generally referred to as relative survival, and we shall do so in this paper. However, we note here that it is a convenient shorthand rather than an accurate description of the calculation method.

While it is very common to take the marginal net survival as a target of estimation, this measure has been noted to have some deficiencies when the aim is to compare net survival between populations or periods of time, including not necessarily being universal due to a dependence on the distribution of risk factors and background mortality rates.^4^ However, in this paper we focus on estimators of the marginal net survival, noting that similar problems will occur in standardised measures of net survival. Both cause-specific and relative survival analysis suffer from potential sources of bias in estimation of the marginal net survival.^5^ Cause-specific survival may be biased by: (1) ascertainment of cause of death, since death certificates are known to suffer from misclassification;^6^ (2) deaths that are indirectly caused by the disease of interest but are not ascribed to the disease (e.g. death from treatment of cancer that would not have occurred if the cancer had not been present);^5,7^ and (3) informative censoring.^8^ Relative survival may be biased if the lifetables for calculation of the expected survival are not representative of the cancer population.^5,9^ For example if: (1) cancer patient mortality from other causes is substantially different from the population mortality, in some cases due to side effects of cancer treatment; (2) other causes of death with shared risk factors (e.g. smoking); (3) cancer patients may be otherwise healthier than average, especially if the cancer is screen-detected; or (4) frailty of cancer patients, in which the more frail patients die of all causes at a faster rate than the general population in the years immediately following diagnosis, but the remaining cancer patients have a lower rate of mortality than the general population thereafter.

Due to the potential for misclassification of cause of death, the current consensus is that relative survival is the preferred method for calculating the marginal net survival for population-based studies, but may not be so in a trial context with review of individual deaths and stringent criteria for the cause of death being from the cancer.^5^ Also, the entry criteria to trials will usually imply that the population under study is not representative of the general population and may therefore have different all-cause mortality rates. While use of relative survival is likely to be the safer option in most cases, in certain circumstances there are possible exceptions to this rule. For example, in lung cancer survival, smoking is a strong confounder for survival from all causes.^10^ In this case, the difference in all-cause hazard of death between lung cancer patients and the general population would be inflated by (for example) heart disease deaths caused by smoking. This suggests as a possible refinement to seek ways of correcting the population hazard for differences in rates of death from specific non-cancer causes between the general population and the cancer cases.

Misclassification of cause of death has been investigated in the context of clinical trials, often trials of screening for cancer.^11–24^ These studies have calculated the sensitivity and specificity of death certificates in reporting specific cancers as the cause of death compared to cause of death assigned by expert review committees (the ‘gold standard’). This raises the question as to whether these error rates could be applied to cause-specific survival to account for misclassification in population-based survival estimates.

The above prompts the question: can we reconcile the two methods and explain the differences? The purpose of this study was to examine the similarities and differences in cancer survival estimates using cause-specific and relative survival methods for a number of malignancies and (1) to transform either to a ‘better’ estimate of net survival by correcting cause-specific survival for misclassification in cause of death using error rates from published clinical trials; and (2) investigate whether the differences suggest a way of using both population hazards and information on cause of death data to further refine relative survival estimates. We note that informative censoring frequently arises, but this does not seem to be an issue with the data used here (but see Discussion below).

## Methods

### Study population

Data on patients diagnosed with a colorectal, lung, breast, ovarian, oesophageal or prostate cancer between 1 January 2006 and 31 December 2015 were extracted from the Cancer Analysis System held by the National Cancer Registration and Analysis Service at Public Health England. Cancers were classified according to the International Classification for Diseases version 10,^25^ colorectal (ICD-10 C18–21, excluding C21.8), lung and bronchus (C33–34), breast (C50, female only), ovarian (C56), oesophageal (C15) and prostate (C61). Demographic and tumour characteristics, including age at diagnosis, sex, year at diagnosis, topography (site of tumour), morphology (type of tumour), income deprivation quintile and geographic location (at government office region level) were extracted. Quintile of income deprivation was based on the local area of residence at the time of diagnosis.^26^

The United Kingdom and Ireland Association of Cancer Registries guidelines on population-based cancer survival analysis^27^ were followed to define the analytical cohort. In short inclusion criteria were: patients aged between 15 and 99 years (inclusive); the earliest primary tumour within the diagnosis period (although the subjects may have had cancers before the period began); and malignant primary tumours. Tumours were excluded if they were missing information on sex, date of diagnosis, date of birth or age; or they were a death certificate only registration. Following the same guidelines, we added one day to the survival of zero-day survivors who were not death certificate only cases, so that they did not enter and exit the at-risk group on the same day. Survival rates are presented for both sexes and all ages from 15 to 99 unless otherwise stated.

Cancer registrations are routinely linked to death registrations held by the Office for National Statistics, enabling access to date of death and cause of death as ascertained from the death certificate.

### Comparison of net survival estimates

Survival time was measured in days and began at date of diagnosis and ended at the first date of death, embarkation or 1 January 2017. Overall and cause-specific survival were estimated using the Kaplan–Meier method. The former considered all deaths as failure events, whilst the latter considered deaths that were attributable to the cancer as recorded on the death certificate as failure events, and all other causes of death were censored. Three different classifications were used to define attribution of cause of death to the cancer of interest whereby the underlying cause of death on the death certificate was (1) the cancer site of interest only, (2) defined using the Surveillance Epidemiology and End Results (SEER) cause-specific death classification, which uses the cancer site of interest, organ system of interest and any related comorbidity,^28^ (3) any cancer. The ICD-10 codes for these rules can be found in Supplementary Table 1.

Relative survival was calculated unstandardised using the Pohar-Perme estimator,^3^ which provides an estimate of net survival in a relative survival framework. The estimates are derived from subtraction of the population hazard of death from that in the cancer patients (see Supplementary Information 1). As noted above, the estimator is not calculated as a relative figure but as a transformation of a difference in hazards, but we retain the terminology of relative survival as a convenient shorthand. Expected survival was calculated using life-tables by age (1-year) and gender based on the English population obtained from the Cancer Survival Group at the London School of Hygiene and Tropical Medicine. We used the command strs in Stata Version 15.1 for the Pohar-Perme estimates.^29^ To investigate the effect of different life-tables on the relative survival estimates, two separate life-tables were applied. The first stratified the English population by age (1-year), sex and year; the second stratified by geographical location (government office region) and deprivation quintile (according to the income domain scores of the Indices of Multiple Deprivation) in addition to age (1-year), sex and year. The case survival data extends to the end of 2016, whereas the population life tables are based on empirical data to the end of 2014 with extrapolation thereafter. To further adjust for deprivation, we used updated life tables from the Office for National Statistics and Public Health England. We have retained the London School of Hygiene and Tropical Medicine life tables for the age/sex adjusted relative survival, for consistency with previous reported survival estimates.

### Sources of error

#### Misclassification in cause of death

In most cases, relative survival is lower than cause-specific.^30^ To explore the extent that this might be explained by errors in cause of death ascertainment, we first identified a number of publications on misclassification in cause of death for persons with specific cancer—from these, we obtained estimates of the sensitivity and specificity of death certificates in reporting specific cancers as the cause of death. The false negative and false positive error rates were obtained from the existing literature if they were based on a cohort of cancer patients only. These error rates were used to correct the cause-specific survival estimates as follows. Algebraically, it is simpler to work with fatality rather than survival estimates. Suppose at a given time that *p* is the true net fatality (complement of the net survival), *p*_2_ the observed cause-specific fatality (complement of the cause-specific survival), *q* the observed all-cause fatality (complement of the overall survival) and *p*_1_ the observed relative fatality (complement of the relative survival). Suppose further that *r* and *s* are the false negative and false positive probabilities of classifying the cause of death as from the cancer of interest. Then the probability of dying of the cancer is *p* and the probability of dying of other causes is (*q* *–* *p)*. The probability of being observed to die of the cancer is

$$p_2 = p\left( {1 - r} \right) + \left( {q - p} \right)s$$

This solves for *p* as:

1 $$p = \frac{{p_2 - qs}}{{1 - r - s}}$$

For our purpose, exploration of sources and magnitude of error, we apply these estimates to all cases. A limitation is that it is likely, however, that errors in cause of death ascertainment will vary by age and other risk factors.

#### Differences in non-malignant chronic disease mortality between cancer patients and general population

The relative survival estimate may be biased if patients with a given cancer have different risks of death from other causes to those in the general population. For example, some risk factors for common cancers, such as smoking, are also risk factors for heart disease. It might be that the appropriate general population risk should be inflated for heart disease deaths, to reflect an increased risk of heart disease death in the cancer patients, due to the common risk factor. We therefore estimated the mortality rates for ischaemic heart disease (IHD) in patients with each of the six cancers by sex for the two age groups 55–64 y and 65–74 y. We then compared these with the corresponding mortality rates in the general population. From the relative rates compared to the general population, we calculated a relative fatality estimate with an approximate correction for a difference in risk of dying of IHD as:

2 $$p_1^ \ast = \left( {1 - h} \right)p_1 + \frac{{hp_1}}{\varphi }$$

where *h* is the proportion of deaths from IHD among the cancer cases, φ is the relative risk of dying of IHD for the cancer cases compared with the general population and $p_1^ \ast$ is the corrected relative fatality. This is a weighted average of the conventional relative fatality and one divided by the relative risk of dying from IHD among the cancer patients (i.e. with the expected fatality adjusted by the relative risk of dying of IHD among cancer patients). This is not a formal correction, which will be the subject of future research, but an ad hoc measure of the potential magnitude of error in relative survival when there is a disparity in risk of death from a relatively frequent cause between the cancers and the general population, and the extent to which this might explain differences between relative and cause-specific survival.

#### Correction of the estimated population hazard for smoking

As already noted, for cancers strongly associated with smoking, the cancer cases will have a higher prevalence of smoking than the general population. We investigated this for lung cancer by estimating the relative rate of all-cause mortality by smoking (ever–never) and the difference in smoking prevalence between lung cancer cases and the general population. This gave a correction to the population other cause hazard. Details are given in Supplementary Information 2. This was done separately for men and women as both relative rates and prevalence varied substantially by sex.

## Results

Table 1 shows overall 10-year survival (death from any cause), cause-specific survival, relative survival using age and sex, and relative survival using age, sex and deprivation, for the six cancers. Results are shown stratified by age and sex. In these cancers, the proportions excluded due to missing data were 1% of cases. The relative survival is usually lower than the cause-specific, with particularly large differences observed for lung cancer, ovarian cancer and colorectal cancer. For prostate cancer, and to a lesser extent breast cancer, the relative survival is higher than cause-specific. These patterns were observed within each age group investigated, except for breast and prostate cancer where relative survival was very slightly lower than cause-specific for the youngest age group (15–44 years). For relative survival, inclusion of deprivation as one of the standardising variables made minor differences to the estimates. For example, in males, the 10-year relative survival estimates for lung, prostate, oesophageal and colorectal cancers were respectively 0.06, 0.79, 0.11 and 0.53 when deprivation was included, compared to 0.06, 0.81, 0.12 and 0.54 when only age and sex were used.

**Table 1:** Ten-year survival estimates by age, sex and cancer type.

Age group	Survival method	Lung		Prostate	Breast	Ovary	Oesophagus		Colorectal	
		Men	Women	Men	Women	Women	Men	Women	Men	Women
15–99 years	All-cause	0.04	0.06	0.52	0.64	0.33	0.08	0.09	0.33	0.35
	Cause-specific	0.10	0.12	0.75	0.80	0.43	0.14	0.14	0.57	0.57
	Relative^a^	0.06	0.08	0.81	0.81	0.39	0.12	0.11	0.54	0.54
	Relative^b^	0.06	0.08	0.79	0.80	0.38	0.11	0.11	0.53	0.54
15–44 years	All-cause	0.22	0.27	0.84	0.79	0.78	0.15	0.23	0.59	0.61
	Cause-specific	0.27	0.33	0.87	0.81	0.82	0.02	0.29	0.67	0.69
	Relative^a^	0.23	0.28	0.86	0.80	0.79	0.16	0.23	0.60	0.62
	Relative^b^	0.22	0.28	0.86	0.80	0.79	0.16	0.23	0.60	0.62
45–54 years	All-cause	0.10	0.15	0.84	0.83	0.56	0.15	0.21	0.52	0.57
	Cause-specific	0.14	0.19	0.89	0.86	0.62	0.20	0.28	0.61	0.66
	Relative^a^	0.10	0.15	0.90	0.86	0.58	0.16	0.21	0.55	0.59
	Relative^b^	0.10	0.15	0.89	0.86	0.58	0.16	0.21	0.55	0.59
55–64 years	All-cause	0.07	0.11	0.78	0.80	0.37	0.13	0.19	0.51	0.56
	Cause-specific	0.12	0.16	0.87	0.86	0.44	0.19	0.25	0.64	0.67
	Relative^a^	0.09	0.12	0.92	0.88	0.40	0.15	0.21	0.60	0.62
	Relative^b^	0.08	0.12	0.91	0.87	0.40	0.15	0.21	0.59	0.61
65–74 years	All-cause	0.05	0.07	0.62	0.67	0.23	0.10	0.13	0.40	0.46
	Cause-specific	0.11	0.14	0.82	0.82	0.32	0.17	0.20	0.62	0.65
	Relative^a^	0.07	0.09	0.93	0.87	0.30	0.14	0.17	0.60	0.61
	Relative^b^	0.07	0.09	0.89	0.83	0.29	0.14	0.17	0.57	0.59
75–99 years	All-cause	0.01	0.02	0.22	0.24	0.08	0.02	0.02	0.14	0.17
	Cause-specific	0.06	0.07	0.55	0.61	0.19	0.07	0.06	0.46	0.45
	Relative^a^	0.04	0.05	0.62	0.63	0.18	0.06	0.04	0.45	0.46
	Relative^b^	0.03	0.04	0.62	0.63	0.17	0.06	0.04	0.46	0.47

^a^adjusted for age, sex and year.

^b^adjusted for age, sex, year, deprivation and government office region.

For cause-specific survival, as one would expect, when a larger range of deaths were considered attributable to the cancer (using SEER cause-specific death classification), estimates were reduced (Table 2). For example, in males, the 10-year cause-specific survival estimates for lung, prostate, oesophageal and colorectal cancers were, respectively, 0.09, 0.74, 0.12 and 0.49, compared to 0.10, 0.75, 0.14 and 0.57 for survival to death from only the cancer of interest. Estimates were similar to the relative survival for oesophageal and ovarian cancers. For lung cancer, estimates were only slightly lower and were still higher than relative survival. For breast and prostate cancer, the difference in cause-specific survival and relative survival was even wider. For colorectal cancer, the cause-specific survival was substantially reduced and was lower than the relative survival. When any cancer death was considered attributable to the cancer of interest, the estimates were further reduced.

**Table 2:** Classification of cause of death on ten-year cause-specific survival^a^.

Age group	Cause of death	Lung		Prostate	Breast	Ovary	Oesophagus		Colorectal	
		Men	Women	Men	Women	Women	Men	Women	Men	Women
15–99 years	Cancer of interest	0.10	0.12	0.75	0.80	0.43	0.14	0.14	0.57	0.57
	SEER classification	0.09	0.11	0.74	0.79	0.39	0.12	0.13	0.49	0.49
	Any cancer	0.07	0.10	0.67	0.76	0.37	0.11	0.12	0.46	0.47
15–44 years	Cancer of interest	0.27	0.33	0.87	0.81	0.82	0.02	0.29	0.67	0.69
	SEER classification	0.24	0.31	0.88	0.80	0.80	0.17	0.24	0.62	0.63
	Any cancer	0.24	0.30	0.87	0.80	0.80	0.17	0.24	0.62	0.63
45–54 years	Cancer of interest	0.14	0.19	0.89	0.86	0.62	0.20	0.28	0.61	0.66
	SEER classification	0.13	0.18	0.87	0.86	0.59	0.17	0.25	0.56	0.60
	Any cancer	0.12	0.17	0.83	0.85	0.58	0.17	0.24	0.55	0.59
55–64 years	Cancer of interest	0.12	0.16	0.87	0.86	0.44	0.19	0.25	0.64	0.67
	SEER classification	0.12	0.15	0.81	0.86	0.41	0.17	0.23	0.59	0.61
	Any cancer	0.10	0.14	0.74	0.83	0.39	0.16	0.22	0.56	0.59
65–74 years	Cancer of interest	0.11	0.14	0.82	0.82	0.32	0.17	0.20	0.62	0.65
	SEER classification	0.10	0.13	0.59	0.81	0.29	0.14	0.18	0.55	0.58
	Any cancer	0.08	0.11	0.51	0.76	0.27	0.13	0.17	0.51	0.54
75–99 years	Cancer of interest	0.06	0.07	0.55	0.61	0.19	0.07	0.06	0.46	0.45
	SEER classification	0.05	0.06	0.24	0.59	0.16	0.05	0.05	0.36	0.36
	Any cancer	0.04	0.05	0.20	0.52	0.14	0.05	0.05	0.31	0.34

^a^Details on ICD-10 codes for each classification can be found in Supplementary Table 1.

Published false negative and false positive rates of classifying death as being from the relevant cancer are given in Supplementary Table 2. Corrected estimates of cause-specific survival for the relevant cancers, using Eq. (1), are shown in Table 3. Cancer of the oesophagus is omitted from this table, as we were unable to find published false positive and negative rates for classification of deaths by oesophageal cancer. For cancers of the breast, prostate, lung and colorectum, there were multiple estimates of errors of ascertainment, and so for these we report two corrections. For lung cancer and ovarian cancer, corrections for errors of ascertainment probably over-correct, in that the corrected survival is lower than the overall survival. For prostate cancer, one correction, where the death certificate cause was defined very narrowly as prostate cancer being the primary cause of death on the certificate, corrected the cause-specific survival estimate for all ages substantially downwards from 0.75 to 0.62 at 10 years (when the relative survival was substantially higher than the cause specific, at 0.81). The other correction for prostate cancer and corrections for breast cancer made little difference to the estimates, and indeed for breast cancer, relative and cause-specific survival rates were very close. For colorectal cancer survival, the corrected 10-year cause-specific survival was slightly lower than the relative survival.

**Table 3:** Ten-year cause-specific survival corrected for misclassification of cause of death.

Age group	Correction applied^a^	Lung		Prostate	Breast	Ovary	Oesophagus		Colorectal	
		Men	Women	Men	Women	Women	Men	Women	Men	Women
15–99 years	No correction	0.10	0.12	0.75	0.80	0.43	0.14	0.14	0.57	0.57
	Correction 1	0.02	0.05	0.75	0.81	0.32	–	–	0.53	0.52
	Correction 2	0.00	0.03	0.62	0.78	–	–	–	0.49	0.49
15–44 years	No correction	0.27	0.33	0.87	0.81	0.82	0.02	0.29	0.67	0.69
	Correction 1	0.21	0.30	0.86	0.80	0.79	–	–	0.63	0.65
	Correction 2	0.19	0.26	0.86	0.78	–	–	–	0.60	0.63
45–54 years	No correction	0.14	0.19	0.89	0.86	0.62	0.20	0.28	0.61	0.66
	Correction 1	0.06	0.12	0.88	0.86	0.54	–	–	0.57	0.62
	Correction 2	0.05	0.11	0.89	0.85	–	–	–	0.54	0.60
55–64 years	No correction	0.12	0.16	0.87	0.86	0.44	0.19	0.25	0.64	0.67
	Correction 1	0.05	0.10	0.87	0.87	0.33	–	–	0.61	0.64
	Correction 2	0.03	0.07	0.87	0.85	–	–	–	0.58	0.61
65–74 years	No correction	0.11	0.14	0.82	0.82	0.32	0.17	0.20	0.62	0.65
	Correction 1	0.04	0.08	0.82	0.83	0.19	–	–	0.59	0.61
	Correction 2	0.01	0.05	0.82	0.81	–	–	–	0.55	0.58
75–99 years	No correction	0.06	0.07	0.55	0.61	0.19	0.07	0.06	0.46	0.45
	Correction 1	−0.04	−0.02	0.53	0.63	0.04	–	–	0.41	0.40
	Correction 2	−0.05	−0.03	0.55	0.57	–	–	–	0.36	0.36

^a^Details of the false negative and false positive error rates used in the corrections can be found in Supplementary Table 2.

Supplementary Table 3 gives the rates of death from IHD in cancer patients and in the general population for males and females and for selected age groups. Rates of IHD death were substantially higher than in the general population for cases of lung, ovarian, oesophageal and colorectal cancers. Rates were substantially lower than the general population for prostate cancer cases and slightly lower for breast cancer. When applied to age-specific 10-year survival (age 55–74) for each cancer using Eq. (2), relative survival remained unchanged for cancers of prostate, breast and ovary. Relative survival was increased slightly for lung cancer, oesophageal cancer and colorectal cancer (Table 4). For colorectal cancer, this was driven by persons aged 65–74 years.

**Table 4:** Ten-year relative survival taking account of difference in deaths due to ischaemic heart disease between cancer population and general population.

Age group	Correction applied^a^	Lung		Prostate	Breast	Ovary	Oesophagus		Colorectal	
		Men	Women	Men	Women	Women	Men	Women	Men	Women
55–74 years	No correction	0.08	0.10	0.92	0.88	0.35	0.15	0.19	0.60	0.62
	IHD correction	0.09	0.11	0.92	0.88	0.35	0.16	0.20	0.61	0.62
55–64 years	No correction	0.09	0.12	0.92	0.88	0.40	0.15	0.21	0.60	0.62
	IHD correction	0.10	0.12	0.92	0.88	0.40	0.16	0.21	0.60	0.62
65–74 years	No correction	0.07	0.09	0.93	0.87	0.30	0.14	0.17	0.60	0.61
	IHD correction	0.08	0.10	0.87	0.87	0.30	0.15	0.18	0.61	0.61

^a^Details of relative risk used in correction can be found in Supplementary Table 3.

The excess and cause-specific hazards by cancer and year are given in Supplementary Table 4. When we corrected the population hazards for smoking in estimation of relative survival in lung cancer (Table 5), we found that the 10-year relative survival (all ages) in men shifted from 0.06 to 0.07, and in women from 0.08 to 0.10.

**Table 5:** Ten-year relative survival corrected for smoking prevalence.

Age group	Correction applied	Lung	
		Men	Women
15–99 years	No correction	0.06	0.08
	Smoking correction	0.07	0.10
15–44 years	No correction	0.23	0.28
	Smoking correction	0.23	0.28
45–54 years	No correction	0.10	0.15
	Smoking correction	0.11	0.16
55–64 years	No correction	0.09	0.12
	Smoking correction	0.09	0.13
65–74 years	No correction	0.07	0.09
	Smoking correction	0.08	0.11
75–99 years	No correction	0.04	0.05
	Smoking correction	0.05	0.07

## Discussion

We found that for cancers of the lung, oesophagus, ovary and colorectum, relative survival was lower than cause-specific. Studies estimating error rates in death certificate cause of death suggest that there can be substantial inaccuracy in cause of death ascertainment, which does not affect the relative survival, but may substantially affect cause-specific survival. In addition, further stratification for deprivation (in case of socioeconomic associations with the cancer) in estimation of relative survival for the cancers considered here tended to make little difference to the survival estimate, or if anything resulted in a further reduction.

Two notable exceptions to the above were breast and prostate cancer. For breast cancer, relative and cause-specific survival were close to each other, with cause-specific survival slightly lower. For prostate cancer, cause-specific survival was considerably lower than relative. Breast and prostate cancer patients might be expected to have better survival to death from other causes than the general population. In the case of breast, this is because breast cancer patients will have slightly higher socioeconomic status on average than the general population. This can be observed in our data, as when the lifetables are also stratified by deprivation, the 10-year survival was the same as the cause-specific survival (moving the relative survival from 0.81 to 0.80). Another reason is that large numbers of breast cancers are screen-detected and women who attend for screening have on average better health status than the general population. In the case of prostate cancer this might be because prostate cancer patients were more health conscious and had sought medical advice for urological symptoms as a result. For prostate cancer, inclusion of deprivation moved the 10-year relative survival from 0.81 to 0.79, towards but still relatively distant from the cause-specific estimate of 0.75. We might expect therefore, that the other cause hazard might be overestimated for these patient groups. This in turn would underestimate the net hazard and therefore overestimate net survival. Thus, we see in practice that in contrast to many other cancers, the net relative survival for breast and prostate cancer is higher than the cause-specific survival. For breast cancer, this phenomenon is no longer observed when socioeconomic status is added to population life tables for calculating net survival. For prostate, the phenomenon is still present, although less extreme, when socioeconomic status is controlled for in the net survival estimation.

The lower relative survival for lung cancer, and higher relative survival for prostate and breast cancers has been observed by others, including in other countries.^31,32^ Withrow et al.^33^ in Canada also observed lower relative survival for lung and colorectal cancer and higher relative survival for breast and prostate cancers. However, it should be noted that some of the other results are based on different methods of calculation of relative survival. For example, Makkar et al.^31^ used the Ederer method rather than Pohar-Perme.

Formal correction for published estimates of error rates in determining cause of death was not generally successful, in that most corrected estimates were not credible. The estimated error probabilities varied substantially among studies. Some of the more extreme estimates were derived from small numbers. For example, the high false positive rates for lung cancer reported by Horeweg et al and Yousaf-Khan et al were based on 8 and 36 deaths respectively.^13,20^ The published estimates were mainly in the context of screening trials carried out some time ago, often decades ago, and errors may be specific to the study population and epoch. No published error rates were available for oesophageal cancer. For lung and ovarian cancers, the resulting estimates were clearly overcorrections, since they gave survival estimates lower than the overall survival. For breast cancer, correction made little difference, but indeed for breast cancer there is little difference between relative and cause-specific survival in any case. For prostate cancer, all published corrections (Table 3) except one made little difference. The one exception was that where a very narrow definition of cause of death from death certification was used,^11^ which gave a very high false negative rate, and corrected the cause-specific survival downwards. That is, the correction increased the distance between relative and cause-specific survival.

Interestingly, for colorectal cancer, the error corrections resulted in a cause-specific survival slightly lower than the relative survival using age, sex and time only. However, two of the error corrections were almost exactly the same as the relative survival estimate including deprivation and another was almost exactly the same as the cause-specific estimate using the SEER cause-specific death classification. This suggests that neither relative survival stratifying only on age, sex and time nor cause-specific survival using primary cause as the cancer site of interest only is accurate as a measure of the burden of excess mortality in colorectal cancer patients. A target for the future is to map the two-dimensional range of false positive and false negative rates which are consistent with the differences between cause-specific and relative survival, specific to particular cancers.

The informal correction for differential rates of IHD death only gave slightly different survival rates for cancers of the lung, oesophagus and colorectum but not for prostate, breast or ovary. The former are all smoking-related cancers, thus one might assume that a large proportion of these patients smoked and would have a higher risk of death due to IHD compared to the general population. The relative risk of death due to IHD needed to be high in order for any change in relative survival to be apparent.

The use of a single overall correction for misclassification of cause of death or IHD death may be too simple. For a given cancer, the difference between relative and cause-specific survival is not uniform by age and sex. For example, the difference between the two estimates for oesophageal cancer is greater for women than for men, and the difference for colorectal cancer is greater for disease diagnosed at younger ages. Thus, it may be that age-sex specific corrections, if they could be estimated, might bring the two survival estimates closer together.

A possible approach to correcting relative survival is to consider that for some cancers, for example, lung, the cases are characterised by high rates of smoking which is also a risk factor for other cancers and non-malignant chronic diseases. This means that some of the excess mortality observed in lung cancer cases is due to deaths from other smoking-related diseases. We therefore corrected the population hazard of death from other causes, used to calculate relative survival, based on the difference in smoking prevalence between the general population and lung cancer patients and the excess all-cause mortality due to smoking. Our results made small differences in unconditional survival estimates, but substantially larger differences in survival conditional on surviving at least one year (results not shown). Thus, this estimation tactic might be generally useful in cancers strongly related to smoking. It should be noted, however, that Hinchcliffe et al.,^34^ in an elegant series of analyses of lung cancer survival, postulating different all-cause mortality relative risks for smoking, found that the bias engendered in the relative survival estimates by the effect of smoking on all-cause mortality was likely to be small.

Another issue with relative survival is the possibility that deaths from the cancer in question form a sufficient proportion of all-cause deaths to inflate the expected numbers of deaths, thus overestimating relative survival. This has been found to be a minor phenomenon for the most part but can cause noticeable bias for cancers which occur predominantly in old age, for example prostate cancer.^35,36^ In our data, relative survival was higher than cause-specific for prostate cancer, partly due to the prostate cancer cases being unrepresentative of the general population, as noted above, but possibly also due to the contribution of prostate cancer deaths to all-cause deaths. This can be particularly seen at ages 75 and over.

Informative censoring is often a major issue, particularly with cause-specific survival. However, in the data considered in this paper, it does not seem to arise. The classic manifestation of informative censoring occurs when cases are accrued over a long period of time, during which the distribution of factors affecting survival changes. A paradigmic example is when due to an ageing population, age at diagnosis is greater on average in more recent cases, so that follow-up time is shorter on average in older patients, who often have poorer survival.^37^ However, we have a relatively short period of case accrual and indeed the opposite phenomenon is seen, that there is more censoring among younger cases. For example, for ovarian cancer, 83% of cases diagnosed at ages 15–44 were censored, whereas in cases aged 75 or over, only 17% were censored. The higher censoring rate for the younger age group is due not to informative censoring but to the fact that the younger cases have better survival.

A target for the future is to find a factor, which accounts for the higher relative survival compared to cause-specific survival in prostate cancer. In this case, further stratification by deprivation increased the discrepancy if anything. There must therefore be other covariates which might account for the difference, bringing cause-specific and relative survival closer together. Mode of detection (symptomatic or screening) would be a sensible target, although this is difficult to ascertain with the health data resources available. However, in many cases, there may be sufficient data to stratify by stage.

It is worth bearing in mind that although one would tend to be reassured by agreement between the two survival measures (for example, the differences for breast cancer are very small), it is at least possible that both are inaccurate, one for reasons of misclassification of cause of death, the other because of confounders such as smoking or the contribution of cancer-specific deaths to all-cause mortality as mentioned above.

The SEER cause-specific death classification^28^ offers a possible method for addressing misclassification in cause of death when calculating cause-specific survival. The classification is defined using information on cause of death as well as number of tumours in a patient (only one or multiple tumours), site of the original cancer (including organ systems) and comorbidities (site-related disease or AIDs and cancer). When used, this classification produces survival estimates similar to that of relative survival for most cancers and could be a potential method when appropriate lifetables are not available for relative survival. For cancers of the lung, ovary and oesophagus, this definition moves the cause –specific survival closer to the relative survival, whereas for prostate, it increases the distance between the two estimates. For colorectal cancer, it results in an estimated survival considerably lower than both relative and cause-specific survival.

For relative survival, we used the Pohar-Perme method, since this is commonly used for routine reporting. However, it can be imprecise, particularly for long term or otherwise low survival, and can give estimates which are not comparable between different cohorts. Use of other net survival estimates, or standardised relative survival may be more appropriate.^4^

The work reported on here is of its nature exploratory. It has shed light on the essential problems but has not provided solutions. We only studied six specific malignancies. Correction of cause-specific survival for misclassification was generally unsatisfactory. In cancers where relative survival might be inaccurate, methodology to correct this has still to be developed.

Our results were consistent with the current consensus that relative survival is generally preferable to cause-specific survival. A priori, cause-specific survival is sensitive to errors in cause of death ascertainment, while relative survival is not. However, we can never be completely certain which is more correct. There are cancers for which relative survival may be inaccurate, in particular those where for demographic (mainly socioeconomic) or lifestyle (particularly smoking) reasons, the other cause mortality rates in the cancer patients differ substantially from those in the general population. Likely solutions to this would be to enhance the life tables used for standardisation, to include other demographic variables than age and sex, and to stratify standardisation by cause of death.

## Supplementary information

Supplementary Information

## Data Availability

Data for this study is based on patient-level information collected by the NHS, as part of the care and support of cancer patients. The data is collated, maintained and quality assured by the National Cancer Registration and Analysis Service, which is part of Public Health England.
