# Supplementary material for: Errors in determination of net survival: cause-specific and relative survival settings
Source: Br J Cancer. 2020 Feb 10;122(7):1094–101. doi: 10.1038/s41416-020-0739-4 (PMC7109046; doi:10.1038/s41416-020-0739-4)
Supplement: Supplementary file 1 — Supplementary Information [file 41416_2020_739_MOESM1_ESM.docx]

**Supplementary Material**

**Errors in determination of net survival: cause-specific and relative survival settings**

CJ Bright, AR Brentnall, K Wooldrage, J Myles, P Sasieni, SW Duffy

Corresponding author: Chloe J Bright. National Cancer Registration and Analysis Service, Public Health England, 2 Rivergate, Temple Quay, Bristol, BS1 6EH. Email: [chloe.bright@phe.gov.uk](mailto:chloe.bright@phe.gov.uk). Tel: 01179689133

Supplementary Table 1. Rules used to define cause of death attributable to cancer of interest*. Codes are based on the International Classification of Disease Version 10 (ICD-10)

| Cancer site | Cancer site of interest only | SEER cause-specific death classification | | Any cancer death |
| --- | --- | --- | --- | --- |
|  |  | Patients with one tumour | Patients with multiple tumours |  |
| Lung | C33-C34 | Any cancer: C00-D489  AIDs and cancer: B21 | Site-specific: C32-C34, C39, C780, D022, D143, D144, D15, D38  Non-site specific: C798, C80, C97, D489 | C00-C97 |
| Prostate | C61 | Any cancer: C00-D489  AIDs and cancer: B21  Site-specific: N40-N50 | Site-specific: C60-C63, D075, D291, D40, N40-N50  Non-site specific: C798, C80, C97, D489 | C00-C97 |
| Breast | C50 | Any cancer: C00-D489  AIDs and cancer: B21  Site-specific: N61-64 | Site-specific: C445, C50, D05, D225, D24, D485, D486, N61-64  Non-site specific: C798, C80, C97, D489 | C00-C97 |
| Ovary | C56, C57.0-C57.7 | Any cancer: C00-D489  AIDs and cancer: B21  Site-specific: N71-N85 | Site-specific: C51-C57, C79, D073, D27, D39, N71-N85  Non-site specific: C798, C80, C97, D489 | C00-C97 |
| Oesophagus | C15 | Any cancer: C00-D489  AIDs and cancer: B21  Site-specific: K20-K31, K51-K57, K92 | Site-specific: C15-C16, C26, D001, D130, D371-D379, K20-K31, K51-K57, K92  Non-site specific: C798, C80, C97, D489 | C00-C97 |
| Colorectum | C18-C20, C21.8 | Any cancer: C00-D489  AIDs and cancer: B21  Site-specific: K20-K31, K35-K38, K51-K57, K62, K63, K65, K66, K92 | Site-specific: C17-C21, C26, C785, D010-D012, D12, D371-D379, K20-K31, K35-K38, K51-K57, K62-K63, K65-K66, K92  Non-site specific: C798, C80, C97, D489 | C00-C97 |

*must appear as underlying cause of death on death certificate as per rules defined by the Office for National Statistics

Supplementary Table 2. Ten-year cause-specific survival corrected for estimates of false positive and false negative probabilities of attributing death to specific cancers

| Cancer | Correction number | Literature source | False negative probability | False positive probability |
| --- | --- | --- | --- | --- |
| Lung | 1 | Yousef-Khan et al, 2017^23^ | 6.60% | 47.70% |
|  | 2 | Miller et al, 2015^20^ | 9.40% | 6.50% |
|  | 3 | Horeweg et al, 2012^16^ | 4.80% | 37.50% |
| Ovary | 1 | Miller et al, 2015^20^ | 16.00% | 1.60% |
| Colorectal | 1 | Doria-Rose et al, 2010^13^ | 10.20% | 4.10% |
|  | 2 | Atkin et al, 2010^24^ | 15.50% | 0.90% |
|  | 3 | Miller et al, 2015^20^ | 10.40% | 1.70% |
| Breast | 1 | Goldoni et al, 2009^14^ | 2.40% | 8.00% |
|  | 2 | Holmberg et al, 2009^15^ | 11.60% | 4.00% |
|  | 3 | Chamberlain et al, 1991^12^ | 3.70% | 19.40% |
| Prostate | 1 | Turner et al, 2016^21^ | 9% | 8% |
|  | 2 | Barry et al, 2013^11^ | ‘First position’ 35.10% | 3.10% |
|  | 3 |  | ‘any position’ 5.40% | 5.80% |
|  | 4 | Makinen et al, 2009^19^ | 3.90% | 1.10% |
|  | 5 | Loffeler et al, 2018^18^ | 14.50% | 20.80% |
|  | 6 | Miller et al, 2015^20^ | 10.90% | 1.70% |
|  | 7 | Walter et al, 2017^22^ | Netherlands 7.00% | 0.90% |
|  | 8 |  | Belgium 6.00% | 0.60% |
|  | 9 |  | Sweden 1.90% | 1.50% |
|  | 10 |  | Finland 3.10% | 2.50% |
|  | 11 |  | Switzerland 7.50% | 6.90% |
|  | 12 | Kilpelainen et al, 2016^17^ | 4.60% | 5.20% |

Supplementary Table 3. Relative risk of death from ischaemic heart disease in cancer patients compared to the general population

| Sex | Age | Lung | Prostate | Breast | Ovary | Oesophagus | Colorectum | Rate of death in Population (per 100,000) |
| --- | --- | --- | --- | --- | --- | --- | --- | --- |
| Male | 55-64 | 6.0 | 0.7 | — | — | 3.8 | 1.7 | 149.4 |
|  |  | (5.3-6.6) | (0.6-0.8) |  |  | (3.1-4.7) | (1.5-1.9) | (148.0-150.7) |
|  | 65-74 | 4.1 | 0.7 | — | — | 2.5 | 1.5 | 353.2 |
|  |  | (3.9-4.4) | (0.7-0.8) |  |  | (2.2-2.9) | (1.4-1.6) | (350.9-355.5) |
|  | 55-74 | 5.4 | 0.9 | — | — | 3.2 | 1.8 | 236.7 |
|  |  | (5.1-5.7) | (0.9-0.9) |  |  | (2.9-3.6) | (1.7-1.9) | (235.6-238.1) |
| Female | 55-64 | 6.7 | — | 0.9 | 1.9 | 4.8 | 1.7 | 38.5 |
|  |  | (5.4-8.2) |  | (0.8-1.1) | (1.3-2.7) | (2.7-8.7) | (1.3-2.2) | (37.8-39.1) |
|  | 65-74 | 4.5 | — | 0.9 | 1.3 | 3.6 | 1.6 | 130.8 |
|  |  | (4.1-5.0) |  | (0.8-1) | (1.0-1.6) | (2.7-4.8) | (1.4-1.8) | (129.4-132.2) |
|  | 55-74 | 6.0 | — | 1.0 | 1.5 | 4.6 | 2.0 | 79.2 |
|  |  | (5.4-6.5) |  | (0.9-1.0) | (1.2-1.8) | (3.5-6.0) | (1.8-2.2) | (78.5-79.9) |
| Persons | 55-64 | 6.1 | — | — | — | 5.0 | 1.9 | 93.1 |
|  |  | (5.6-6.8) |  |  |  | (4.2-6.1) | (1.7-2.1) | (92.3-93.8) |
|  | 65-74 | 4.4 | — | — | — | 3.3 | 1.7 | 237.5 |
|  |  | (4.2-4.7) |  |  |  | (2.9-3.7) | (1.6-1.8) | (236.2-238.9) |
|  | 55-74 | 5.7 | — | — | — | 4.2 | 2.0 | 156 |
|  |  | (5.4-5.9) |  |  |  | (3.8-4.6) | (1.9-2.1) | (155.3-156.7) |

*Based on 11 cases

Supplementary Table 4. Ten-year excess and cause-specific hazards for six malignancies^1^

| Time since diagnosis | Lung | | Prostate | | Breast† | | Ovary | | Oesophagus | | Colorectum | |
| --- | --- | --- | --- | --- | --- | --- | --- | --- | --- | --- | --- | --- |
|  | Excess | Cause-specific | Excess | Cause-specific | Excess | Cause-specific | Excess | Cause-specific | Excess | Cause-specific | Excess | Cause-specific |
| 1 | 1.10 | 1.00 | 0.04 | 0.05 | 0.04 | 0.04 | 0.32 | 0.27 | 0.87 | 0.79 | 0.28 | 0.22 |
| 2 | 0.50 | 0.46 | 0.03 | 0.04 | 0.03 | 0.03 | 0.17 | 0.15 | 0.54 | 0.50 | 0.12 | 0.11 |
| 3 | 0.28 | 0.25 | 0.03 | 0.03 | 0.03 | 0.03 | 0.13 | 0.12 | 0.28 | 0.25 | 0.08 | 0.07 |
| 4 | 0.19 | 0.16 | 0.02 | 0.03 | 0.03 | 0.03 | 0.09 | 0.09 | 0.16 | 0.15 | 0.05 | 0.05 |
| 5 | 0.13 | 0.10 | 0.02 | 0.03 | 0.02 | 0.02 | 0.07 | 0.06 | 0.10 | 0.09 | 0.03 | 0.04 |
| 6 | 0.10 | 0.08 | 0.02 | 0.02 | 0.02 | 0.02 | 0.05 | 0.05 | 0.06 | 0.06 | 0.02 | 0.02 |
| 7 | 0.09 | 0.06 | 0.01 | 0.02 | 0.02 | 0.02 | 0.04 | 0.03 | 0.04 | 0.04 | 0.01 | 0.02 |
| 8 | 0.08 | 0.05 | 0.01 | 0.02 | 0.01 | 0.01 | 0.03 | 0.03 | 0.04 | 0.03 | 0.01 | 0.01 |
| 9 | 0.07 | 0.04 | 0.02 | 0.02 | 0.01 | 0.01 | 0.02 | 0.02 | 0.03 | 0.02 | 0.01 | 0.01 |
| 10 | 0.08 | 0.04 | 0.01 | 0.02 | 0.01 | 0.01 | 0.03 | 0.02 | 0.03 | 0.02 | 0.00 | 0.01 |

^1^ details of how the average hazards for each year were calculated are provided in the Supplementary methods

† Females only

**Supplementary information 1: Average hazard calculation**

Throughout the manuscript we have compared survival estimates. In addition we thought it may be informative to compare the excess hazards to the cause-specific hazards. Details on how this was calculated are shown below.

The relationship between the hazard $h_{T}\left( t \right)$ and survival $S_{T}\left( t \right)$ functions can be shown as

$S_{T}\left( t \right)=exp[-H_{T}\left( t \right)]$ (1)

where

$$H_{T}\left( t \right)= \int_{0}^{t} h_{T}\left( t \right)dt$$

The average hazards for each year can be calculated as

$h_{T}\left( t \right)- h_{T-1}(t)$

Using the relationship in equation 1 we can use our survival estimates (Table 2) to calculate the average hazard per year as

$-ln[{S_{T}\left( t \right)}/{S_{T-1}\left( t \right)}]$ (2)

**Supplementary information 2: Adjustment for smoking to the population hazards used to calculate relative survival from lung cancer**

Because large numbers of lung cancer patients are smokers or ex-smokers, these patients will have higher all-cause mortality rates than the general population, and therefore the estimated background mortality hazard rates used to calculate relative survival should be adjusted for this. We adjust the hazards for males and females separately, as relative risks and proportions of smokers and ex-smokers vary between the sexes.

For males, we take the relative risks of all-cause mortality by smoking status from the British Doctors’ Study, males born from 1900 onwards. Relative risks of 1.00, 1.31 and 2.19 were observed for never-, ex- and current smokers respectively [1]. Office of National Statistics Publications report that in male general population subjects, 56% are never-smokers, 27% ex-smokers and 17% current smokers [2]. In lung cancer patients in the Liverpool Lung Project, 5% were never-smokers, 46% ex-smokers and 49% current smokers [3]. The overall proportions are given here, as within the lung cancer patients the distributions were almost identical between the sexes. Thus, if h represents the rate of death from all causes in never smokers, the expected rate in the general population would be

$$g=h\times(0.56\times1.00+0.27\times1.31+0.17\times2.19)=1.29\times h$$

and in lung cancer patients the rate would be

$$l=h\times(0.05\times1.00+0.46\times1.31+0.49\times2.19)=1.73\times h$$

Thus the lung cancer patients would be expected to have a hazard 34% higher (1.73/1.29=1.34) than the general population. We therefore inflated the other cause hazard for male lung cancer patients by 34%.

For females, the general population proportions of never, ex and current smokers are 63%, 24% and 13%. [2]. We used data from the Million Women Study for the relative risks of all-cause mortality associated with smoking [4], 1.00, 1.27 and 2.62 for never-, ex- and current smokers respectively. This gives an expected rate in the general population of

$$g=h\times(0.63\times1.00+0.24\times1.27+0.13\times2.62)=1.28\times h$$

For female lung cancer cases, the rate would be

$$l=h\times(0.05\times1.00+0.46\times1.27+0.49\times2.62)=1.92\times h$$

Thus the female lung cancer patients would have a 50% higher hazard of death from other causes than the general female population (1.92/1.28=1.50), and we therefore inflated the other cause hazard for female lung cancer patients by 50%.

**References**

1. Doll R, Peto R, Boreham J, Sutherland I. Mortality in relation to smoking: 50 years’ observations on male British doctors. BMJ 2004; 328: 1519
2. <https://www.ons.gov.uk/peoplepopulationandcommunity/healthandsocialcare/healthandlifeexpectancies/bulletins/adultsmokinghabitsingreatbritain/2017>
3. Cassidy A, Myles JP, van Tongeren M, Page RD, Liloglou T, Duffy SW, Field JK. The LLP risk model: an individual risk prediction model for lung cancer. Br J Cancer 2008; 98: 270-76
4. Pirie K, Peto R, Reeves GK, Green J, Beral V. The 21st century hazards of smoking and benefits of stopping: a prospective study of one million women in the UK. *Lancet* 2013; **381**(9861): 133-141
